# Bibliometric analysis and global trends in uterus transplantation

**DOI:** 10.1097/JS9.0000000000001470

**Published:** 2024-04-17

**Authors:** Tong Wu, Yangyang Wu, Kebing Nie, Jinfeng Yan, Ying Chen, Shixuan Wang, Jinjin Zhang

**Affiliations:** a National Clinical Research Center for Obstetrical and Gynecological Diseases, Tongji Hospital, Tongji Medical College, Huazhong University of Science and Technology; b Key Laboratory of Cancer Invasion and Metastasis, Ministry of Education, Tongji Hospital, Tongji Medical College, Huazhong University of Science and Technology; c Department of Obstetrics and Gynecology, Tongji Hospital, Tongji Medical College, Huazhong University of Science and Technology, Wuhan; d School of Materials Science and Engineering, Huazhong University of Science and Technology, Wuhan; e College of Animal Science and Technology, Sichuan Agricultural University, Sichuan, People’s Republic of China

**Keywords:** absolute uterine factor infertility, bibliographic, Mayer–Rokitansky–Küster–Hauser, uterus transplantation, vascularized composite allotransplantation

## Abstract

**Aim:**

The purpose of this study was to characterize publication patterns, academic influence, research trends, and the recent developments in uterus transplantation (UTx) across the globe.

**Methods:**

The Web of Science Core Collection database was searched for documents published from the time the database began to include relevant articles to 15 December 2023. With the use of VOSviewer, Citespace, BICOMB, and Incites, a cross-sectional bibliometric analysis was conducted to extract or calculate the evaluative indexes. Publications were categorized by country, institution, author, journal, highly cited papers, and keywords. The variables were compared in terms of publication and academic influence, which further included citation count, citation impact, Hirsh index, journal impact factor, total link strength, collaboration metrics, and impact relative to the world.

**Results:**

A total of 581 papers concerning UTx were initially identified after retrieval, and 425 documents were included. Of the 41 countries participating in relevant studies, the USA and Sweden were in leading positions in terms of publications, citations, and academic influence. The most versatile institution was the University of Gothenburg, followed by Baylor University. The most productive scholars and journals were Brännström M. and *Fertility and Sterility*, respectively. Five groups of cutting-edge keywords were identified: venous drainage, donors and donation, women, fertility preservation, and fertility. Topics about surgery, first live birth, risk, and in vitro fertilization remain hot in this field.

**Conclusions:**

UTx is anticipated to enter a golden era in the coming years. This study provides some guidance concerning the authors involved in promoting UTx research, the current development of UTx, and journals to submit their innovative research. This also helps to reach a comprehensive insight and prospect in the near future. In order to establish recognized standards and benefit more patients who are disturbed by uterine infertility, large-scale and well-designed clinical trials are required.

## Introduction

Highlights

- This study is the first thorough bibliometric analysis of uterus transplantation, which is the only choice for absolute uterine factor infertility.
- The USA and Sweden were in leading positions in terms of publications, citations, and academic influence.
- The most productive scholar was Brännström M. from the University of Gothenburg, and the most productive journal was *Fertility and Sterility*.
- The cutting-edge keywords were clustered into five groups: venous drainage, women, fertility preservation, fertility, as well as donors and donations.

Absolute uterine factor infertility (AUFI) is a major type of female infertility that primarily results from the congenital absence of the uterus, previous hysterectomy, and severe intrauterine adhesion. It is estimated to affect ~100 million fertile-age women around the globe^1^. Historically, women with AUFI have had limited options for motherhood and are restricted to adoption and surrogacy. However, adoption does not establish a genetic relationship between the child and the mother, and surrogacy is not permitted in numerous jurisdictions. In addition, these alternatives may introduce legal, cultural, ethical, and emotional challenges for the family. As a result, novel approaches are desperately needed to address AUFI and advance female fertility.

Since the beginning of the 21st century, uterus transplantation (UTx) has emerged as the only treatment option for AUFI. The Turkish first pregnancy and Swedish first live birth of a healthy baby after UTx demonstrated the feasibility of childbirth via UTx, which inspired reproductive centers across the world^2,3^. Following this breakthrough, similar cases have been reported in the USA, Brazil, France, and China^4–7^. The progression of UTx has been further propelled by innovative advancements such as the introduction of robotic-assisted surgeries, the establishment of the International Uterus Transplantation Society, and the identification of the outflow utero-ovarian veins. However, UTx is still in its infancy compared to other solid organ transplantations, with just over 80 UTx operations performed in nearly 20 centers globally by 2023^8,9^. As an innovative surgical method, UTx is worth considerable attention, especially in the context of an aging population and continuously declining fertility rates.

**Figure 1:**
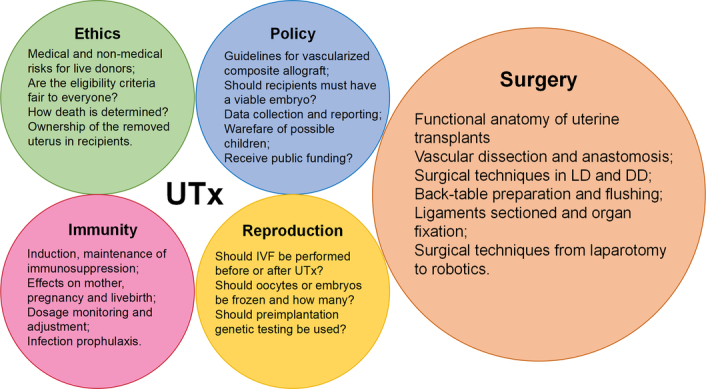
Multidisciplinary characteristics of human uterus transplantation (UTx) and questions in each research areas that are in need of additional evidence to address. DD, deceased donor; IVF, in vitro fertilization; LD, live donor.

With regard to ethics^10,11^, policy^12,13^, surgical methods^14,15^, and immunity (Fig. 1)^16^, UTx is a complex clinical practice. Although UTx treatment has been reviewed from these aspects, there has been little examination of previous publications, academic influence, and research trends in UTx across the globe. Bibliometric methods have been used to explore the productivity of countries, institutions, and researchers within a given subject area and to identify remarkable events, papers, and future trends. Therefore, by using bibliometric analysis, the publication and researcher performance were examined. It helps experts and newcomers to identify the breadth of their field, locate fresh topics of interest, and develop future research plans visually.

## Methods

The institutional review board of the Tongji hospital deemed that ethical approval was not necessary. This study is a cross-sectional observation study, and thus, we followed the reporting guidelines of the Strengthening the Reporting of Observational Studies in Epidemiology^17^. It helps us uphold the highest standards of scientific rigor, objectivity, and replicability characteristic of our manuscript.

### Data collection

We used the Web of Science Core Collection (WOSCC) to identify publications of UTx from the time the database began to include relevant articles to 15 December 2023. In order to encompass all the literature pertaining to the research topic while excluding irrelevant documents as much as possible, we conducted a topical search using the query: ‘uterus transplantation’ or ‘uterine transplantation’ and human. Retrieved papers were exported with full records and cited references in plain text format.

### Selection of eligible studies

**Figure 2:**
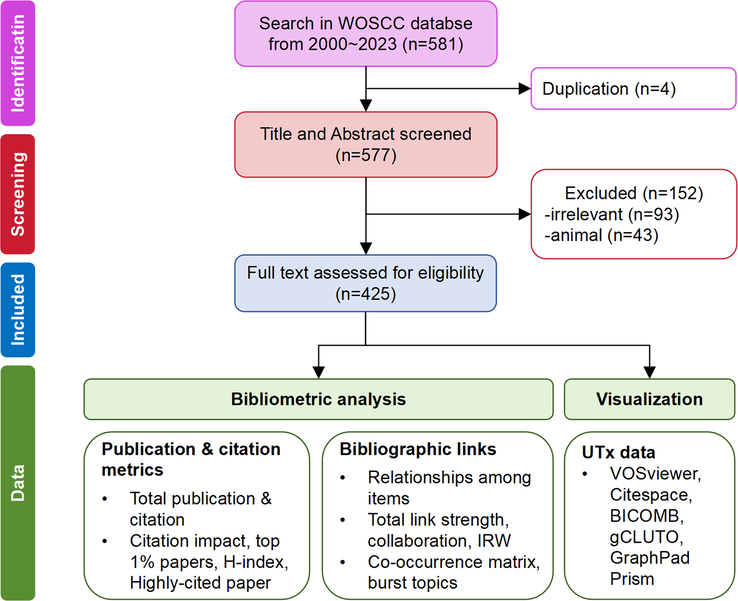
Flow diagram of study selection and data analysis strategies. H-index, Hirsh index; IRW, impact relative to the world; WOSCC, Web of Science Core Collection.

The ‘plain text’ was imported into EndNote X9 for deduplicate. The procedure for data retrieval and collection is shown in Figure 2. It is necessary to conduct a manual screening process in order to identify the final literature to be analyzed. Therefore, the authors (T.W. and Y.Y.W.) independently screened the title and abstract to identify the available studies based on the following criteria. Inclusion criteria were the following: (i) papers focusing on human UTx; and (ii) language was limited to English. Exclusion criteria were the following: (i) irrelevant topics that superficially mentioned UTx (e.g. studies that investigated disorders lead to AUFI but not treatment); (ii) studies conducted only on animal models and no human experiments or work have been conducted; and (iii) papers with missing information (e.g. author details, journal name, or publication year). We set no restrictions for study design (e.g. observational, experimental) or type of publication (e.g. editorial, original article, review). Discrepancies between selected studies by both authors were discussed in a consensus meeting, with the senior author (J.J.Z.) giving a binding verdict.

### Data extraction, standardization, and analysis

The VOSviewer 1.6.18 was used to cluster the countries/regions, institutions, and journals. The Citespace 6.2.4 was used for visualizing the authors’ network, generating keyword maps, and conducting burst word analysis. Most importantly, synonymous expressions were standardized manually to eliminate redundant entries. For instance, ‘uterus transplantation’ and ‘uterine transplantation’ were merged. The BICOMB 2.0 was used to perform a co-occurrence matrix of high-frequency keywords, and then gCLUTO was used to generate a matrix visualization and a mountain visualization. In addition, the GraphPad Prism 8.0 was used to perform the statistical analyses. The country’s level of income was determined according to World Bank data for 2023.

### Academic influence measurement

Academic influence is a master criterion for assessing the academic excellence of the document, journal, institution, researcher, etc., and its measurement is inherent and diverse. We used the combined metrics to measure academic influence (Table 1).

**Table 1:** Indications for academic influence metrics in this study.

Metric	Indication
Citation count	Number of times a country/institution/author/journal is cited
Citation impact	Average number of citations a document received
Hirsh index (H-index)	A researcher has an h-index if they have at least h publications for which they received at least h citations
Journal impact factor (JIF)	functional approximation of the mean citation rate per citable item
Total link strength	Total strength of the co-authorship links of a given country/institution/author with others
Domestic collaborations	Papers with two or more distinct addresses and all addresses in the same country
International collaboration	Papers that contain one or more international co-authors
Impact relative to the world	Citation impact of the set of publications as a ratio of the world average
Highly cited paper (HCP)	Top one percent in each of the Essential Science Indicators subject areas per year

## Results

### Baseline characteristics of the eligible documents

**Figure 3:**
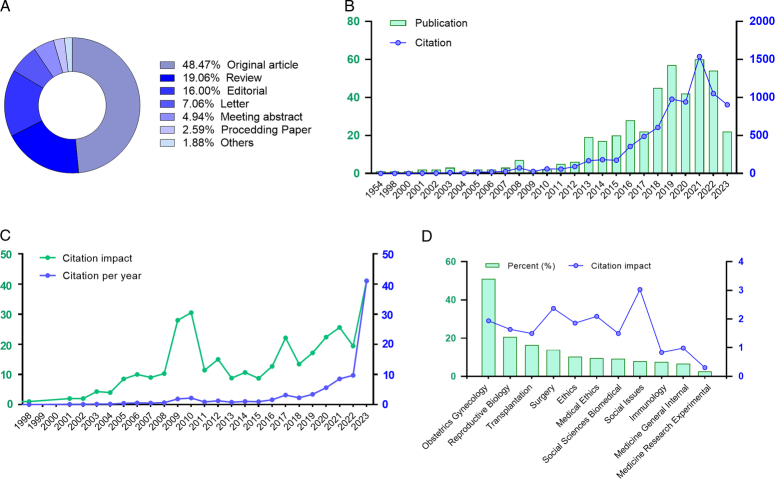
Baseline bibliometric characteristics of all eligible documents. (A) Pie chart showing the article types of the 425 papers. (B) Annual publications and citations, as well as (C) citation impact and citation per year about UTx. No data was produced in 1954 because the paper received no citation. (D) Document percent and citation impact according to the research areas of human UTx studies.

A total of 581 publications were initially retrieved from the search (Fig. 2). After excluding studies that were duplicated, irrelevant, and restricted to animal models (*n*=152), a total of 425 documents were identified as eligible studies. These documents encompassed several types of publications, with original articles constituting 48.47% and reviews accounting for 19.06% (Fig. 3A). Moreover, 16.00% of the publications included editorials, indicating that there is a recognized significance for UTx. Interestingly, UTx received little attention until 2013, with no more than six papers published annually (Fig. 3B). However, the number of publications per year has shown a consistent increase since 2013 and will reach a peak of 60 publications in 2021. A similar pattern was also observed in the number of citations, which began to increase in 2016 and peaked at 1537 in 2021 (Fig. 3B). The citation impact displayed a slightly increasing trend from 2001, with a noticeable increase in 2009 and 2010 (Fig. 3C). When adjusted for the year, the citation per year demonstrated a continuous increase in the past 5 years (Fig. 3C). With regard to the involved research areas, over half of the papers (*n*=217, 51.06%) belonged to the Obstetrics Gynecology (OB/GYN) category, followed by Reproductive biology (*n*=88, 20.71%), Transplantation (*n*=70, 16.47%), Surgery (*n*=59, 13.88%), and Ethics (*n*=44, 10.35%) (Fig. 3D). However, the most citations were given to social issues and surgery (with an impact of 3.0 and 2.4, respectively), indicating their consistent public interest. Taken together, UTx is an interdisciplinary field involved in medicine, ethics, and sociology that is developing rapidly at the moment.

### History of UTx

**Figure 4:**
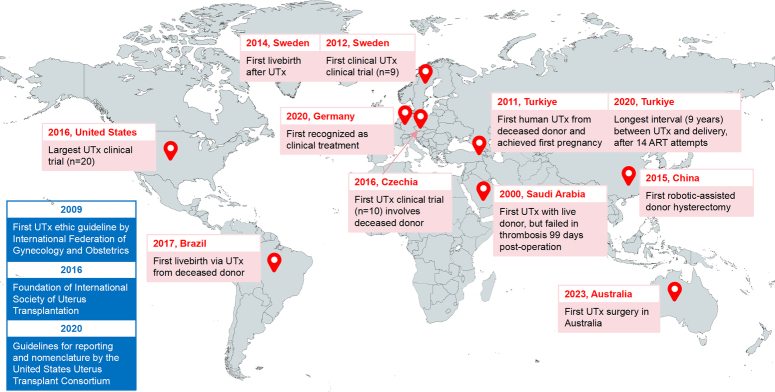
History and milestone of human UTx. Years and countries of most first events and encouraging achievements were shown. ART, assisted reproductive technology.

There are some chronological key events in the history of UTx that promoted its development since its inception (Fig. 4). In April 2000, after performing UTx in baboons and goats, Fageeh *et al*.^18^ in Saudi Arabia reported the first human UTx, which ended in the removal of a necrotic uterus 99 days later. They concluded that UTx is feasible and safe and that, to implement the discipline in human clinical practice, surgical, vascular anatomy, and strong fixation are necessary. However, there was a 10-year silence on UTx following the first case. Until 2011, surgeons from Turkiye performed another UTx operation using a donated uterus from a brain-dead, 22-year-old female^19^. The woman achieved the first pregnancy after embryo transfer, but ended in chemical pregnancy^3^. Afterward, clinical trials involving live donors (LDs) or deceased donors (DDs) were approved and initiated^2,5,20^. UTx completed the mission for AUFI in 2014. For the first time, a live birth was reported after UTx in Sweden, and it provided detailed references on patient selection, surgical steps, immunosuppression strategy, etc^2^. Although cesarean delivery was undertaken in 32 weeks, the first postnatal week of the newborn was uneventful, indicative of being normal for gestational age. The first live birth following UTx from a DD was reported in a patient with Mayer–Rokitansky–Küster–Hauser syndrome in Brazil^21^. In 2015, Dr Wei at Xijing Hospital in China performed the world’s first robot-assisted donor surgery. The operation took only 6 h, which is much shorter than previous studies^6^. In January 2016, the International Society of Uterus Transplantation was founded and comprised of world experts and pioneers dedicated to the development of UTx. In recent years, UTx or live births have been achieved in many countries or regions, and the processes were similar to those of prior studies.

### Distributions of countries

**Figure 5:**
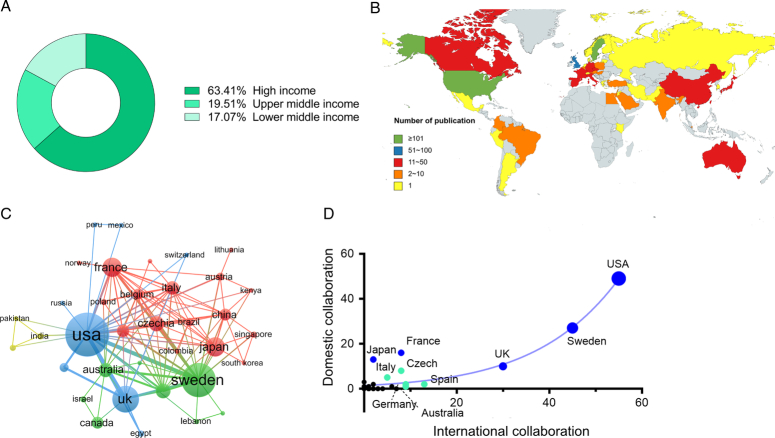
Relationship and cluster of countries. (A) Classification of countries according to the income levels. (B) Geographical UTx activity based on the number of publications. (C) The relationship between international and domestic collaborations. (D) Cooperative relationships among various countries.

Forty-one countries contributed to the global discourse, of which 63.41% were high-income countries, 19.51% were upper-middle-income countries, and 17.07% were lower-middle-income countries (Fig. 5A, Table 2). The geographical distribution of the global productivity map showed that most research publications on UTx originated from European, North American, and Asian nations. The involved countries were grouped into four clusters, indicating diverse regional distribution patterns, possibly stemming from the collaborative relationships among these nations (Fig. 5B). As a result of their high productivity levels, citations, and H-index, the USA and Sweden have been identified as the leading contributors to UTx research (Fig. 5C). The USA had the highest international (value=55), domestic (value=49), and industry collaborations (value=1), highlighting the extensive nature of its collaborative efforts (Fig. 5D). Spain registered the highest indicator (value=4.28) in terms of impact relative to the world, followed by Australia (value=3.96).

**Table 2:** Top 10 productive countries in the uterus transplantation (UTx) field.

Country	Income	Paper	Citation	Citation impact	Top 1% papers	H-index	Total link strength	International collaboration	Domestic collaboration	Industry collaboration	Impact relative to the world
USA	High	167	2913	17	5	30	93	55	49	1	0.97
Sweden	High	101	3282	33	5	31	83	45	27	0	1.74
UK	High	61	1623	27	2	19	49	30	10	0	1.38
France	High	33	383	12	2	11	29	8	16	0	0.65
Japan	High	32	324	10	0	11	12	2	13	1	0.51
Czechia	High	20	250	13	0	9	20	8	8	0	0.66
Spain	High	18	1404	78	1	12	41	13	2	0	4.28
Australia	High	17	1041	61	2	8	25	9	1	0	3.96
Italy	High	16	184	12	2	8	21	5	5	0	0.64
Germany	High	13	222	17	0	7	27	9	2	0	0.98

### Participants of institutions, authors, and highly cited papers

**Figure 6:**
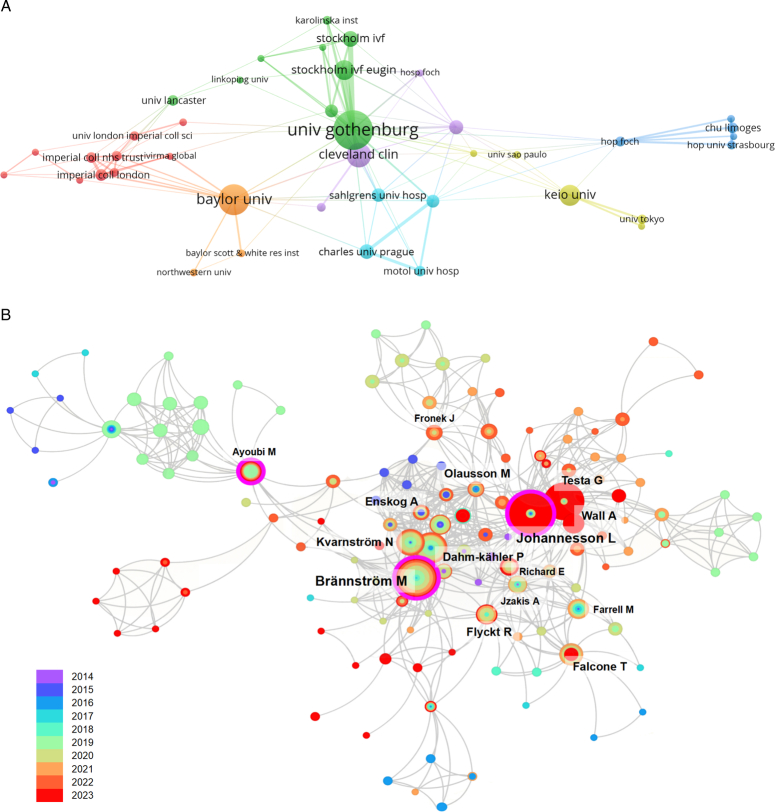
Visualization of the institutions and authors. (A) Collaboration network of the top 40 institutions. (B) Network showing the authors’ relationships. Each circle represents an author, and the different colors represent the years.

The top 10 institutions that published the most papers on human UTx are presented in Table 3. With the highest number of publications (*n*=85) and citations (*n*=2818), the University of Gothenburg in Sweden distinguished itself as an influential contributor to scientific research. The VOSviewer revealed that the University of Gothenburg and Baylor University were representative institutions in their respective countries (Fig. 6A). Moreover, these two organizations worked closely with both domestic and foreign academic institutions. Among the 1083 authors included, the most prolific authors in the bibliometric analysis were Brännström M. (*n*=73), Johannesson L. (*n*=66), and Testa G. (*n*=46) (Table 4). Brännström M. and Johannesson L. had equally strong link strength with other authors (value=180). They were also the top two researchers who cooperated with international and domestic partners. Olausson M. (value=3.28) and Kvarnström N. (value=3.90) were the top two in terms of the impact relative to the world. Both of them worked at the University of Gothenburg, the same as Brännström M. The centralized author clustering suggested close collaborations among these authors (Fig. 6B). Table 5 shows the top 10 original articles that received the most attention. These highly cited papers (HCPs) are sourced from *Fertility and Sterility* (*n*=5), the *Lancet* (*n*=2), the *American Journal of Transplantation* (*n*=2), and the *International Journal of Gynecology & Obstetrics* (*n*=1). All HCPs were published before 2019, aligning with the expected trend that older papers have had more time to accumulate citations than recent ones. The first clinical outcomes of UTx in different aspects have been reported in most studies. For instance, Brännström M. *et al*.^2^ documented the first live birth through UTx, accumulating 500 citations. Notably, the HCPs were predominantly produced by the aforementioned top institutions and authors in the field.

**Table 3:** Top 10 institutions ranked by the counts of publications.

Institution	Country	Paper	% of 425	Citation	Citation impact	H-index	Total link strength
University of Gothenburg	Sweden	85	20.09	2818	33.15	30	104
Baylor University	USA	53	12.53	877	16.55	15	37
Stockholm IVF Eugin	Sweden	44	10.40	1658	37.68	19	36
Cleveland Clinic Foundation	USA	43	10.17	844	19.63	13	23
Sahlgrenska University Hospital	Sweden	39	9.22	1212	31.08	19	17
Keio University	Japan	30	7.09	302	10.07	10	17
Imperial College London	UK	26	6.15	539	20.73	13	31
Harvard University	USA	20	4.73	240	12.00	8	24
Hospital Foch	France	19	4.49	207	10.89	9	27
Charles University Prague	Czechia	17	4.02	233	13.71	8	25

**Table 4:** Top 10 most productive authors ranked by the numbers of publications.

Author	Institution	Paper	Citation	Citation impact	H-index	Total link strength	International collaboration	Domestic collaboration	Impact relative to the world
Brännström M.	University of Gothenburg	73	2618	36	27	180	29	22	1.96
Johannesson L.	Baylor University	66	2159	33	22	180	24	24	1.76
Testa G.	Baylor University	46	858	19	15	104	11	21	1.10
Dahm-Kähler P.	University of Gothenburg	37	1690	46	17	150	15	18	2.39
Kisu I.	Keio University	29	302	10	10	62	2	12	0.53
Kvarnström N.	University of Gothenburg	24	1446	60	14	118	9	12	3.28
Falcone T.	Amer Soc Reprod Med	23	403	18	10	43	4	4	0.89
Olausson M.	University of Gothenburg	23	1794	78	16	111	20	3	3.90
Smith R.	University of New Hampshire	22	495	23	12	34	16	6	1.13
Banno K.	Keio University	21	152	7	7	55	2	5	0.37

**Table 5:** Top 10 cited original articles.

Title	Journal and year	Citation	Main contribution
Live birth after uterus transplantation	Lancet, 2015	500	Clinical course of the first patient who delivered a baby via UTx
Transplantation of the human uterus	International Journal of Gynecology & Obstetrics, 2002	363	The first human UTx confirmed the feasibility and safety of this procedure. But a hysterectomy was conducted after 3 months because of an acute vascular thrombosis
First clinical uterus transplantation trial: a six-month report	Fertility and Sterility, 2014	302	Report about the perioperative and 6-month postoperative outcome of the nine live donor UTx with a low-dose immunosuppressive protocol
Live birth after uterus transplantation from a deceased donor in a recipient with uterine infertility	Lancet, 2018	176	The first live birth following UTx from a deceased donor
Preliminary results of the first human uterus transplantation from a multiorgan donor	Fertility and Sterility, 2013	167	The first long-lived transplanted human uterus with acquirement of 12 menstrual cycles
First live birth after uterus transplantation in the United States	American Journal of Transplantation, 2018	147	The first live birth in the USA with improvements in using utero-ovarian veins and shorter embryo transfer awaiting time
Uterus transplantation trial: 1-year outcome	Fertility and Sterility, 2015	131	Evaluation of long-term uterine viability and function after live donor UTx
Clinical pregnancy after uterus transplantation	Fertility and Sterility, 2013	128	The first clinical pregnancy after UTx
Living Donor Uterus Transplantation: A Single Center’s Observations and Lessons Learned from Early Setbacks to Technical Success	American Journal of Transplantation, 2017	115	In-depth analysis and lessons learned from every UTx, regardless of positive or negative outcomes
One uterus bridging three generations: first live birth after mother-to-daughter uterus transplantation	Fertility and Sterility, 2016	102	The first reporting a live birth after mother-to-daughter UTx, and it also represented the second birth ever after human UTx

### Descriptions of the source journals

**Figure 7:**
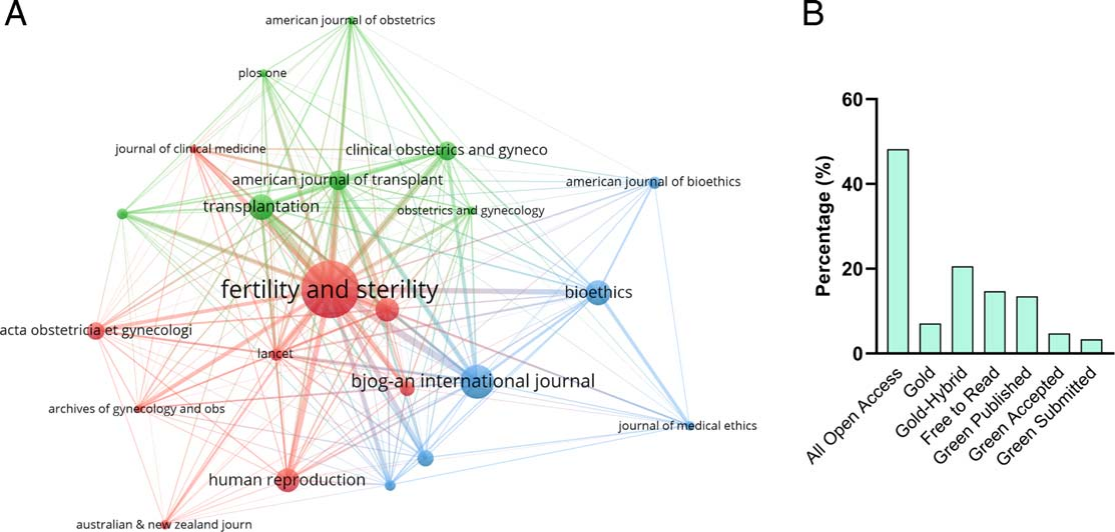
Participants of journals in human UTx. (A) Relationship of the representative journals. The number of links indicated the impact of scholarly publications. (B) Distributions of the open access types.

The top 3 academic journals out of 121 were *Fertility and Sterility* (*n*=48; Table 6), *BJOG – An International Journal of Obstetrics and Gynaecology* (*n*=26), and *Transplantation* (*n*=19). The journal with the highest impact factor was the *Lancet* (JIF=168.9), followed by the *American Journal of Bioethics* (JIF=13.4). Moreover, these relevant journals were categorized into three groups based on their co-citation relationships (Fig. 7A). Leading scientific publisher Wiley published 6 of the top 14 journals, while Elsevier Science Inc. and Lippincott Williams & Wilkins accounted for 3 journals each. More than half of the journals (8/14) fell into the Q1 category, and three Q4 journals had corresponding low H-indexes. Remarkably, none of the journals published relevant papers before 2004. On the other hand, *Fertility and Sterility* has consistently demonstrated productivity in UTx since 2013, while all UTx papers published in *Clinical Obstetrics and Gynecology* were published in 2023. In addition, 50% of all journals were open access, with gold access accounting for 20% (Fig. 7B). The presence of more OA (open access) papers could benefit the field by offering greater visibility and attracting more readers. Overall, these findings demonstrate that journals from various publishers, countries, quartile categories, and access choices actively participated in the research on UTx.

**Table 6:** Top journals ranked by the counts of publications.

Journal	Publisher	Circulation	Open access	Paper	Citation	JIF	Journal citation report	H-index	Total link strength
Fertility and Sterility	Elsevier	195	74.87%	48	1701	6.7	Q1	21	665
BJOG – An International Journal of Obstetrics and Gynecology	Wiley	182	27.07%	26	334	5.8	Q1	8	233
Transplantation	Lippincott Williams & Wilkins	261	8.96%	19	324	6.2	Q1	9	266
Bioethics	Wiley	94	29.48%	18	260	2.2	Q2	9	151
Current Opinion in Organ Transplantation	Lippincott Williams & Wilkins	75	3.44%	17	188	2.2	Q3	6	214
Human Reproduction	Oxford University Press	232	23.04%	17	189	6.1	Q1	6	135
American Journal of Transplantation	Wiley	190	72.50%	14	588	8.7	Q1	10	304
Clinical Obstetrics and Gynecology	Lippincott Williams & Wilkins	72	1.28%	13	23	1.5	Q4	3	189
Acta Obstetricia et Gynecologica Scandinavica	Wiley	141	40.27%	12	194	4.3	Q1	6	96
Journal of Gynecology Obstetrics and Human Reproduction	Elsevier	166	31.93%	11	44	1.9	Q4	3	114
Journal of Obstetrics and Gynaecology Research	Wiley	368	5.58%	10	60	1.6	Q4	3	109
American Journal of Bioethics	Taylor & Francis	20	21.51%	8	100	13.4	Q1	5	50
Lancet	Elsevier	223	26.22%	8	717	168.9	Q1	6	211
International Journal of Gynecology & Obstetrics	Wiley	440	17.89%	7	470	3.8	Q2	5	114

The circulation only contains reviews and articles.

JIF, journal impact factor.

### Keywords of research hotspots

**Figure 8:**
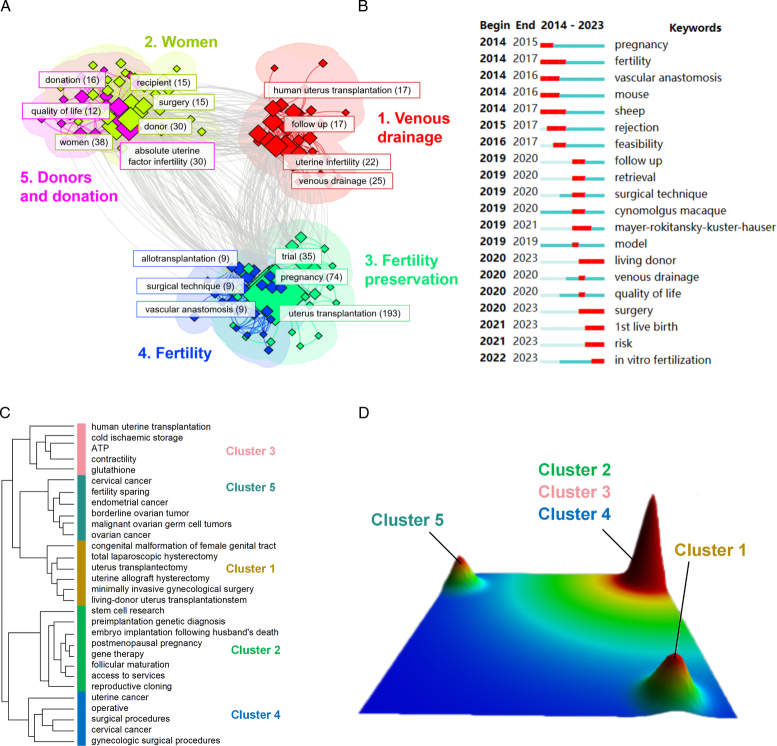
Analysis of keyword co-occurrence. (A) Clustering co-occurrence map of all keywords of studies on UTx. (B) Top 25 keywords with the strongest citation bursts. Matrix visualization (C) and mountain visualization (D) of bi-clustering of the key keywords. The row labels denote high-frequency keywords. The height of the mountain is proportional to the similarity within the cluster.

Keywords play a critical role in reflecting the central messages, areas of interest, and future directions of a discipline. Overall, 980 key terms were retrieved from the titles and abstracts. Table 7 shows the top 20 keywords, which identify established themes such as uterus transplantation, pregnancy, and infertility, as well as uncharted territory, including follow-up, Montreal criteria, and quality of life within the field of study. Figure 8A displays the keywords grouped into five clusters, each represented by a different color. It revealed the importance of the vascular anastomosis procedure (venous drainage), donor selection and surgery (women, and donors and donation), and fertility strategies (fertility preservation and fertility). This study demonstrates further the strength of keyword bursts as a crucial indicator of the study frontiers. Most significantly, the citation burst time of terms such as ‘living donor’ (2020–2023), ‘surgery’ (2020–2023), ‘1st live birth’ (2021–2023), ‘risk’ (2021–2023), and ‘in vitro fertilization’ (2021–2023) persisted into 2023, which indicates the ongoing recognition and interest in these areas (Fig. 8B). Figure 8C shows the visualization matrix of the high-frequency keywords. Mountain visualization was used to verify the effect of the visualization matrix, with each mountain representing a cluster (Fig. 8D).

**Table 7:** Top 20 keywords with the highest frequency related to the human UTx through Citespace.

Keyword	Count	Centrality	Keyword	Count	Centrality
uterus transplantation	193	0.34	donation	16	0.07
pregnancy	74	0.20	recipient	15	0.06
women	38	0.21	deceased donor	15	0.09
trial	35	0.13	living donor	15	0.02
absolute uterine factor infertility	30	0.04	surgery	15	0.01
donor	30	0.05	in vitro fertilization	13	0.04
venous drainage	25	0.05	Montreal criteria	13	0.03
uterine infertility	22	0.11	infertility	13	0.09
follow-up	17	0.11	assisted reproductive technique	13	0.07
human uterus transplantation	17	0.06	quality of life	12	0.05

## Discussion

### Global performance in the human UTx

The current cross-sectional bibliometric analysis performed the status and trends in human UTx research. The results showed that in the last 10 years, particularly since the first live birth following UTx, there has been a significant increase in attention to UTx, and it is experiencing the best of times. It is perhaps that UTx is unique in permitting LD transplantation and for reproductive use. Overall, 425 papers in this field were identified, and 14 milestone events were summarized. Despite the overall volume of research being low, as UTx progressed, more countries are believed to have live births via UTx over time. In particular, the USA, which has contributed the highest number of publications, and Sweden, which has garnered the most citations, have developed a competitive environment. However, there is a decreasing trend in the number of studies from these two countries, while research output from other nations is on the rise. Brännström, M. from the University of Gothenburg, has become the most prolific publisher in the field, and he has also collaborated with other institutions, contributing to the first UTx cases in the Middle East and Australia^22,23^.

### Multidisciplinary research status of UTx

The OB/GYN type accounts for most publications and many more researchers are engaged in UTx from the OB/GYN standpoint. This predominance is justified because UTx is not merely a surgical procedure but a long-term medical management that also covers various OB/GYN aspects, such as in vitro fertilization, gynecological surgery, embryo transfer, and pregnancy monitoring. Notably, multidisciplinary researchers must work together to further enhance UTx. Johannessona *et al*.^14^ suggested that any center implementing a program for UTx should have an established abdominal transplant program, a gynecologic surgery program, a high-risk obstetric and neonatal care, and an institutional support and oversight. Therefore, a successful UTx program requires broad support from various specialties starting with initial patient referral to delivery and follow-up of the child.

In addition to clinical factors, key unresolved issues related to ethics, policy, and immunity also need to be addressed (Fig. 1). For example, who owns and controls the transplanted uterus before and after UTx? As the graft is a transitory organ, it will be removed again after one or two live births. And then, to whom does the uterus belong since it may in theory be implantable in another recipient?^24^ Another important question involves the motivation for patients with AUFI to undergo UTx rather than choose adoption or surrogacy. It could have to do with the cost, adoption qualification, privacy, experience of gestation, and psychological desire for a biological relationship^25^.

### Country-level productivity and collaboration

There are currently no reports of UTx studies conducted in any low-income countries. This is in line with the observation that the poorest third of the world’s population receives only 3.5% of the estimated 234 million major surgical operations undertaken worldwide^26^. The lack of access to surgical care and the lack of surgical necessity, which also applies to UTx, is a result of this vast decline in low-income countries. Barriers may exist in, but are not limited to, the requirement for highly skilled surgical procedures, insufficient research funding, lack of research infrastructure, shortage of awareness, and traditional beliefs about disease processes^27^. Currently, achieving UTx in resource-limited settings is challenging. It is important to strengthen the general healthcare level and basic surgical system in these regions^28^.

The USA and Sweden are the most active countries in the field, with their dominance attributable to their bold innovation and practices, as reflected by a number of initial clinical reports on various aspects of UTx. Other factors contributing to the development of UTx include lower labor costs, fewer regulatory barriers, and a greater number of potential participants. However, there is an ongoing debate regarding whether UTx should be conducted, particularly given that altruistic surrogacy is legally permitted in certain regions^29^. UTx is expected to serve a wider demographic, including transgender male-to-female individuals and cis-gender males. Such cases are more likely to initially emerge in Western countries due to cultural and policy differences. Interestingly, the development of UTx in some countries necessitates support from the top-cited countries. The first UTx Czech clinical trial was in collaboration with Olausson, M., who ranked eighth among the top productive authors^30^. The first live birth following UTx in the Middle East was a result of Swedish–Lebanese–Jordanian cooperation^23^. Since 1998, the Swedish and Australian teams have established a close relationship, and this contributed to the first Australian UTx procedure^22^.

According to the results of an online survey, 62.4% of healthcare providers had heard of UTx, most of whom had heard about it through social media or a news source, but only 17.9% of providers agreed that UTx was relevant to their clinical practice^31^. Professional continuing medical education on UTx is therefore necessary and beneficial. The Swedish team has provided support and training for teams planning UTx procedures internationally^22^. A systematically organized and effective continuing medical education curriculum should encompass qualified faculty, definitive objectives, and methodological details, among other components^32^. Furthermore, telesurgery has emerged as a potential alternative that may advance UTx surgery and provide benefits including reduced costs, enhanced performance, and convenience^33^. In the meantime, the formation of the International Society of Uterus Transplantation and the ethical and reporting guidelines are great approaches to promote the communication and development of UTx.

### Evolutionary trend of HCPs

The top two HCPs reported the first live birth resulting from UTx and the first case of UTx, respectively. However, the former was published in the *Lancet* (JIF=168.9), while the latter appeared in the *International Journal of Gynecology & Obstetrics* (JIF=3.8). This significant gap in JIF could be linked to a potential publication bias against negative results prevalent at that time^34^. Negative trials, however, are equally important since they provide insights into the shortcomings of a procedure. Consequently, the first UTx operation holds equal scientific significance because it underscored the importance of uterine fixation, prompting subsequent improvements to address concerns about compromised blood supply^35^. Similar to this, the first pregnancy after UTx was achieved in a 21-year-old female by Ozkan’s team, but the patient experienced five failed pregnancies afterward^19^. An obstructed blood outflow was observed under the perfusion computed tomography, and so a revision surgery was conducted^3^. This is the first case in which additional revision surgery resulted with a live birth, and it also stressed the important of a thorough imaging examination.

### Discussion on the study of research hotspots

Keyword analysis elucidates the current frontiers in the human UTx field and reflects a trajectory that is in tandem with the rapidly advancing age. This landscape is sculpted by emergent technological innovations and shaped by the demands of an increasingly deserved fertility preservation.

### Vascular anastomosis

**Figure 9:**
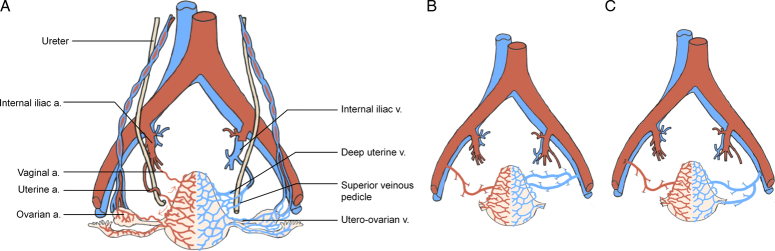
Human uterine graft with arterial and venous anastomoses. (A) Schematic picture of the vasculature network of the female reproductive tract. (B) Both uterine arteries are anastomosed to the recipient’s external iliac arteries end-to-side. The recipient’s uterine veins are anastomosed to the recipient’s external iliac vein end-to-side. (C) The utero-ovarian veins are anastomosed end-to-side to a uterine vein. a. artery; v. vein.

The inflow and outflow of the grafts are established via several uterine arteries and venous pedicles, respectively. Arterial anastomosis is performed with or without anterior division of the internal iliac artery to the external iliac artery of the recipient end-to-side (Fig. 9). In terms of veins, the uterine veins or utero-ovarian veins of the LD graft are anastomosed either separately to the external iliac veins in an end-to-side fashion or the utero-ovarian veins are anastomosed to the graft’s uterine veins, which are anastomosed together to the external iliac veins. With a DD graft, uterine veins are usually anastomosed using a segment/patch of the internal iliac veins^36^. The procured venous pedicles of the first UTx case were elongated by segments of the great saphenous vein and anastomosed to the external iliac veins. Using finer 8–0 Prolene sutures instead of running 7–0 Prolene is another way to improve vein drainage^5^. In addition, a Doppler probe can be secured to one of the uterine arteries to monitor flow in the immediate postoperative period.

### Venous drainage

Correct and efficient venous drainage is necessary to produce a competent uterus that does not show signs of venous congestion, thus facilitating the complete survival of the transplanted tissues. Much drainage of the uterus relies on the deep and superficial uterine veins, with the ureter as the boundary. The former consists of two to six veins on each side and further forms plexuses and ends in the internal iliac vein^37,38^. Dissecting the deep uterine veins is, however, the most demanding part of LD hysterectomy because these veins may be located both ventrally and dorsally to the ureter, making it difficult to procure the graft without damaging the ureter, tiny veins, and nervous plexuses^39^. Poor preoperative imaging and great interindividual and intraindividual variations in number and caliber between and among individuals may cause the disease to worsen. Nevertheless, it is still feasible to obtain long vascular pedicles, including part of the internal iliac veins, without compromising postoperative recovery in LDs (Fig. 9)^40^. Another solution is to divide one of the uterine veins first and reanastomose the divided veins at the back table^40,41^.

Complete utero-ovarian veins may also serve as venous outflow. As demonstrated by Testa *et al*., the use of utero-ovarian veins was safer and easier (Fig. 9). Some teams have also performed UTx with drainage achieved exclusively using utero-ovarian veins and/or ovarian veins^6,42,43^. A normal pregnancy could be sustained solely with utero-ovarian veins, regardless of the uterine veins. It should be noted that bilateral oophorectomy should not be performed in cases of preterm ovarian insufficiency in premenopausal LD. A hazard ratio of 1.41 for all-cause mortality in women younger than 50 years with versus without bilateral oophorectomy was reported. However, there have been reports of uterine and ovarian vein retrievals (including oophorectomies) from premenopausal LD, which led to primary ovarian insufficiency^6,43^.

### Donors and donations

Deceased donation is the standard of care to overcome organ shortage in most developed countries. Most notably, it excludes the surgical risks of LDs, costs less time and energy, and provides optimal uterine vessels (Table 8). Rapid and less extensive donor screening shall generally be carried out within 24 h of the UTx procedure in the event of a planned DD procedure. The ureter can be directly transected in DDs, and the procurement duration is shorter than that in LDs. Internal iliac vessels, along with uterine vessels, are dissected caudally from the common iliacs. All branches from the internal iliacs, except the uterine vessels, are ligated and divided^9^. In 2011, the first UTx from a DD was conducted in Turkeyi, which resulted in pregnancy, but the gestation ended in miscarriage^20,44^. There are controversies with regard to the timing of uterine procurement in relation to the procurement of other organs. If the uterus is excised as the first organ, the cold ischemia times of the other grafts are extended^45^. The uterus can also be retrieved at the very end of a multiorgan retrieval, and this would vice versa prolong the cold ischemia time of the uterine graft^46^.

**Table 8:** Current summary of uterus transplantation trials and cases.

				Surgery					
Author	Country	Starting year	Donor	Method	Duration	Technical success	Blood loss	Hospital stay	Live birth
Brännström *et al*.	Sweden	2012	9	LD (*n*=9): laparotomy	11 h 37 min±1 h 5 min	7/9 (78%)	920±730	6.7±1.6	9
				R (*n*=9): laparotomy	4 h 46 min±30 min	—	670±380	6±0	
Richards *et al*.	USA	2016	8	DD (*n*=5)	—	6/8 (75%)	—	—	1
				R (*n*=10): laparotomy	—		—	—	
Fronek *et al*.	Czech Republic	2016	10	LD (*n*=5): laparotomy	5 h 53 min±42 min	4/5 (80%)	367±418	8±2	3
				DD (*n*=5)	6 h 46 min±65 min	3/5 (60%)	—	—	
				R (*n*=10): laparotomy	4 h 11 min±40 min	—	550±347	12±4	
Testa *et al*.	USA (DUETS)	2016	20	LD (*n*=13): laparotomy	—	8/13 (62%)	—	—	14
				LD (*n*=5): robotic	—	5/5 (100%)	—	—	
				DD (*n*=2)	—	1/2 (50%)	—	—	
				R (*n*=20): laparotomy	—		—	—	
Brucker *et al*.	Germany	2016	4	LD (*n*=4): laparotomy	10.19 h±1.42 h	4/4 (100%)	100	12.75±1.5	2
				R (*n*=4): laparotomy	6.2 h±1.48 h	-	262±160	16±1.83	
Puntambekar *et al*.	India	2017	4	LD (*n*=4): laparoscopy	3.5 h±0.64 h	4/4 (100%)	100	7±1.16	
				R (*n*=4): laparotomy or laparoscopy	4.13 h±0.25 h		400±294	15±6.35	
	Sweden	2017	8	LD (*n*=8): robotic	11.25 h (10-13)	6/8 (75%)	125	5.5 (5–6)	1
				R (*n*=8): laparotomy	5.15 h (4.5-6.6)		300	6 (5–9)	
Fageeh *et al*.	Saudi Arabia	2000	1	LD: laparotomy	—	0	—	—	0
				R: laparotomy	—		—	—	
Ozkan *et al*.	Turkiye	2011	1	DD	2 h	1 (100%)	—	—	1
				R: laparotomy	5.5 h		—	—	
Wei *et al*.	China	2015	1	LD: robotic	8 h	1 (100%)	100	—	1
				R: laparotomy	6 h 50 min		490	1 month	
Ejzenberg *et al*.	Brazil	2016	1	DD	—	1 (100%)	—	—	1
				R: laparotomy	10 h 30 min		800	>8	
Akouri *et al*.	Lebanon	2018	1	LD: laparotomy	10 h 3 min	1 (100%)	900	7	1
				R: laparotomy	6 h 3 min		700	7	
Ayoubi *et al*.	France	2019	1	LD: robotic	13 h	1 (100%)	150	11	
				R: laparotomy	6.5 h		200	11	
Carmona *et al*.	Spain	2020	1	LD: robotic	10 h	1 (100%)	—	4	1
				R: laparotomy	—		—	7	
Vieira *et al*.	Brazil	2021	1	LD: robotic	6 h 40 min	1 (100%)	—	2	
				R: laparotomy	4 h		—	5	
Scollo *et al*.	Italy	2020	1	DD	—	1 (100%)	—	—	1
				R	—		—	—	
Deans *et al*.	Australia	2023	1	LD: laparotomy	9 h 54 min	1 (100%)	—	8	
				R: laparotomy	6 h 12 min		—	8	
Georgevsky *et al*.	Australia	2023	1	LD: laparotomy	9.5 h	1 (100%)	750 ml	8	
				R: laparotomy	4 h		600 ml	7	

### Surgical technique

The classical surgical technique of uterine procurement in LDs is laparotomy, whereas conventional laparoscopy and robotic-assisted laparoscopy are gradually transforming transplantation surgeries. Minimally invasive surgeries are commonly used for hysterectomy, lymphadenectomy, and myomectomy within the field of gynecological surgery. The first case via minimally invasive surgery in UTx included a fully robotic retrieval of the uterus within an LD UTx trial, where a premenopausal mother donated her uterus to her daughter with Mayer–Rokitansky–Küster–Hauser syndrome. Similar to the advantages possessed by traditional robotic surgery, it also shows improvements in terms of three-dimensional optics, camera stability, absence of tremors, and expedited learning curves during UTx^47,48^. Furthermore, using robotics in donor UTx reduces the operation duration (4 h compared to 14 h in the Swedish cases), which allows for greater precision in complex dissection procedures. Taken together, robotic-assisted laparoscopy is a promising technique for donor hysterectomy during UTx. It should be noted that this is a robotic surgical procedure in its infancy and that surely developments will take place within the coming years. These advancements will most likely lead to better outcomes for LDs and recipients. As previously mentioned, it is quite likely that in the relatively near future, most LD UTx cases will be performed by robotic-assisted surgery in both donors and recipients.

### Strengths and limitations

This study has several strengths. First, to the best of our knowledge, this study is the first bibliometric analysis to comprehensively describe the up-to-date development of UTx. It helps OB/GYN scholars to understand the pioneering groups, collaboration patterns, and journal performance in the UTx domain^49^. Second, this study employed fractional counting instead of full counting, which facilitates the normalization of fields and accounts for the confounding effects arising from the aggregation of substantial studies. Third, the assessment of journal performance is not confined to the conventional JIF metric, which solely reflects the journal’s numerical value rather than that of its individual articles. Other metrics, including the H-index, JIF, and total link strength, have also been included for a more comprehensive assessment.

Certain limitations have also been noted in this study. First, it is important to acknowledge that our analysis was solely based on the WOSCC, but this question is inherent to any bibliometric approach. Although this database is comprehensively updated daily and contains a large volume of literature, we are confident that the results achieved in this study are a true reflection of the UTx literature. Comparing results from other databases could be useful for future research; however, this was outside the scope of our current study. Second, although we used the Journal Citation Reports categories to define research fields, these categories do not classify studies in detail. For example, OB/GYN includes a very broad range of highly cited studies. A more detailed classification may be appropriate for a closer look at research trends. Third, while our study identified trends in UTx research, it is important to recognize that it offers a broad overview rather than an in-depth analysis. Several intriguing aspects were not thoroughly explored, including the optimal timing of assisted reproductive technology in relation to UTx and the evolution of vascular anastomosis techniques. These aspects demand further research to provide a more comprehensive understanding of the field.

## Conclusion

UTx is expected to enter a golden era in the coming years after improvements in vascular anatomy, surgical experience, and minimally invasive surgery. It has learned a lot from advancements in the fields of reproductive medicine, transplantation, obstetrics, and gynecology. Most principles of the latter are well established, and minor modifications are required to apply standard practice in UTx. Additionally, UTx has gained global collaboration and has met new challenges.

## Ethical approval

Not applicable.

## Consent

Not applicable.

## Sources of funding

This work is financially supported by the grants from the National Key Research and Development Program of China (No. 2022YFC2704100), and the National Natural Science Foundation of China (Nos. 82301849, 82371648).

## Author contribution

T.W.: wrote the original manuscript and revised, provided grant; Y.W.: wrote the original manuscript and collected literature search; K.N.: collected literature search and performed bibliometric analysis; J.Y.: revised the manuscript; Y.C.: provided design improvement; S.W.: project administration, provided grant, and supervised the study; J.Z.: provided design improvement and grant and revised the manuscript.

## Conflicts of interest disclosure

The authors declare no conflicts of interest.

## Research registration unique identifying number (UIN)

Not applicable.

## Guarantor

Jinjin Zhang had full access to all of the data in the study and take responsibility for the integrity of the data and accuracy of the data analysis.

## Data availability statement

This is a review article, which does not include a data statement.

## Provenance and peer review

Not commissioned, externally peer-reviewed.
